# A Novel Microwave Staring Correlated Radar Imaging Method Based on Bi-Static Radar System

**DOI:** 10.3390/s19040879

**Published:** 2019-02-20

**Authors:** Bo Yuan, Yuanyue Guo, Weidong Chen, Dongjin Wang

**Affiliations:** Key Laboratory of Electromagnetic Space Information, Chinese Academy of Sciences, University of Science and Technology of China, Hefei 230027, China; yuanb@mail.ustc.edu.cn (B.Y.); wdchen@ustc.edu.cn (W.C.); wangdj@ustc.edu.cn (D.W.)

**Keywords:** microwave staring correlated radar, bi-static Radar, inner-and-inter pulse frequency hopping signal

## Abstract

The stochastic characteristic of the radiation field of a mono-static microwave staring correlated imaging (MSCI) radar degenerates with the increase of the imaging distance, which results in degradation of the image quality. To address this issue, a novel MSCI method based on bi-static radar is proposed from two perspectives: site-deploying and waveform design. On the one hand, a new bi-static MSCI site-deploying scheme is proposed which adopts two transmitting stations with their azimuth angles relative to the center of the imaging region differing by 90 degrees. On the other hand, by using two transmitting arrays synchronously transmitting inner-and-inter pulse frequency hopping (IAIP-FH) signals, the radiation field of each station includes a few “frequency stripes” perpendicular to the radiation direction, and as a consequence, the “frequency stripes” of each radiation field are perpendicular to each other. As a result, the radiation field of the bi-static MSCI is the superposition of the two striped radiation fields, thus a latticed radiation field is constructed. Therefore, the targets in different latticed grids scatter independent fields, then, the images can be reconstructed using correlation process (CP) algorithms. The grid size of the latticed radiation field is determined by the inner-pulse frequency hopping (FH) interval of the IAIP-FH signals and the imaging geometry. Moreover, it is shown that the 3 dB beam width of the space correlation function of the radiation field does not change with the imaging distance, thus the stochastic characteristic of the radiation field is partly preserved when the imaging distance increases. Simulation results validate the analysis and show that the proposed method can obtain higher resolution images than the common mono-static MSCI method.

## 1. Introduction

The radar imaging technique has the ability of working all-day and in all weathers [1,2], thus it has attracted increasing attentions and extensive researches, among which earth observation is an important application field [3].

The conventional high-resolution radars for earth observation commonly employ the Range-Doppler (RD) principle [4] or tomography theory [5] to acquire two-dimensional high-resolution images, e.g., synthetic aperture radar (SAR) [6,7,8], etc. For either the RD principle or tomography theory, the azimuth resolution is determined by the aspect-angle variation range during the target observation, thus the radar platforms have to observe the imaging area from multiple observation angles. Therefore, the high-resolution radars based on the two theories all aim to increase the aspect-angle variation range, e.g., spotlight SAR [9,10] and wide-angle SAR [11], and so on. For earth observation, the radar platforms take a long time to obtain multiple sets of observation data from different viewing angles, which greatly increases the time cost and the system complexity.

In many applications, it is necessary to observe a fixed important region continuously using radar systems carried on stationary platforms, for example in important area observations and disaster monitoring. This is called radar staring imaging. In these applications, there is no relative movement between the radar platform and the imaging area, thus the SAR technique cannot be applied. Furthermore, the azimuth resolution of the conventional staring imaging radar, e.g., real aperture radar (RAR), is limited by the aperture size of the antenna array. When the imaging distance increases, the resolution becomes lower. Hence, it is necessary for RAR to use a large-scale antenna array or a sufficient number of stations to form a larger aperture, though this is not always achievable in practice. Therefore, seeking a high-resolution radar imaging method in staring imaging geometry is a hot and difficult problem [12,13].

Recently, microwave staring correlated imaging (MSCI) was proposed as a new microwave staring imaging method, and since then has attracted increasing attentions [14,15,16] due to its ability to acquire high-resolution images in staring imaging geometry.

**Figure 1:**
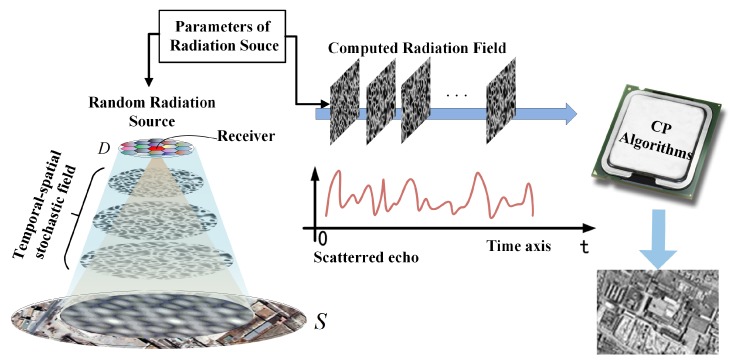
The imaging geometry and procedure of mono-static microwave staring correlated imaging (MSCI).

The detailed imaging process of mono-static MSCI is illustrated in Figure 1. The crucial purpose of MSCI is to construct a temporal–spatial stochastic radiation field (TSSRF) which is realized by random radiation source (RRS), thus the MSCI system commonly consists of multiple transmitters and one or more receivers [14,15,17]. Multiple antennas of the transmitters constitute an array of RRS. First, by using the RRS to transmit predesigned waveforms, the TSSRF is formed at the imaging plane. Then, the receive antenna receives the scattered echo, and after the down-conversion and sampling procedure, the sampled signal is obtained. Finally, the TSSRF matrix is computed according to the parameters of the RRS and the images are reconstructed by correlation process (CP) of the sampled signal and the TSSRF matrix.

The TSSRF in a different spatial position is independent, thus the targets in different locations within the beam coverage scatter independent time-varying fields. Therefore, the MSCI can achieve super-resolution images of the targets. As a consequence, MSCI is superior to RAR and can be used in stationary/quasi-stationary platforms.

The key to MSCI is to construct the TSSRF [14], and the image resolution of MSCI is determined by the stochastic characteristics of the TSSRF, which is heavily dependent on the RRS [14,15,16] design. Therefore, many researchers have studied the RRS design from different aspects. These studies mainly focus on two aspects: (1) multi-channel waveform design, (2) element and layout design of the radiation array.

In waveform design, several commonly used radar waveforms are studied in references [14,15,17,18]. References [14,15] both proposed employing a white Gaussian noise (WGN) signal as the transmitting signal and analyzed its resolution capability. Reference [18] analyzed the usage of the WGN signal in MSCI from a compressive sensing aspect, and pointed out that the column correlation of the sensing matrix of MSCI decreases as the array aperture size increases when the imaging geometry and the size of spatial grids are fixed. However, an ideal WGN signal cannot be easily achieved in engineering due to the bandwidth limitation. In consequence, Reference [17] proposed using inner-pulse frequency hopping (FH) signals which can be easily realized in radar systems and an outfield experiment was performed. However the frequency code design of the FH signal is still an open problem. Reference [19] considered minimizing the condition number of the radiation field matrix and a stochastic optimization algorithm was adopted to acquire a good frequency code design of the FH signal. Contrarily, Reference [20] proposed a method to acquire an ideal orthogonal radiation field by elaborately choosing the waveform parameters. As a consequence, for each pulse, an inverse radiation problem has to be solved to obtain the waveform parameters of each channel. The number of ideal orthogonal radiation fields is finite as a result of the limitation of the bandwidth and the aperture size.

In addition to the waveform design, optimizing the element design and the layout of the array elements can also improve the stochastic characteristic of the TSSRF. Reference [21] proposed the temporal–spatial distribution entropy (TSDE) as the optimization target to optimize the elements’ layout. It is shown that the effective rank of the TSSRF matrix increases as the TSDE increases. Nevertheless, all of the aforementioned researches only utilize the antenna as the basic element of the RSS. Recently, Reference [22] introduced metamaterial apertures [23,24,25,26] into the MSCI, and proposed employing the secondary scattering of a meta-surface to achieve a better radiation field. The meta-surface consists of many evenly spaced complementary-electric-inductor-capacitor (cELC) elements and the scattering property of each cELC element differs from each other. By randomly adjusting the parameters of each cELC, the random radiation field can be achieved. However, using secondary scattering will lead to energy loss and the resolution is still limited by the size of the metamaterial aperture.

The aforementioned researches all focus on the mono-static MSCI system, that is, the transmitting array and the receiver are in the same area. For the mono-static MSCI method, the stochastic characteristic of the TSSRF can be improved using the above methods. However, these methods cannot overcome the problem that the stochastic characteristic of TSSRF degenerates as the imaging distance increases, which lead to the degradation of the imaging results.

Multi-static radars observe the targets from more and larger aspect angles, thus have the potential to form TSSRF with a better stochastic characteristic. Nevertheless, the resolution of MSCI is not determined by the aperture size but by the stochastic characteristic of the TSSRF, and multi-static radar systems greatly increase the system cost and complexity. Therefore, a bi-static radar system rather than a multi-static radar system is considered in this paper. As far as we know, there are no researches on bi-static or multi-static MSCI.

A novel MSCI method based on bi-static radar is proposed which considers both site-deploying and waveform design. First, for site-deploying, the proposed bi-static imaging geometry requires that the azimuth angles of the two transmitting station differ by 90 degrees relative to the imaging area center. Second, every transmitter transmits a IAIP-FH signal to form a “frequency stripe” field with the stripes perpendicular to the radiation direction at the imaging plane. Summing up the above, the radiation field at the imaging plane is the superposition of the two “frequency stripe” fields, thus a latticed radiation is achieved; and so the field in the different lattices is independent and the targets in different lattices scatter independent fields . Finally, high-resolution images can be obtained using the CP algorithms. The grid size of the latticed field is determined by the inner-pulse FH interval of the IAIP-FH signals and the imaging geometry, and is not influenced by the imaging distance.

The remainder of this paper is outlined as follows. The imaging model of the proposed imaging geometry and the waveform design are given in Section 2. In Section 3, the space correlation function of the TSSRF of the proposed method is analyzed. In Section 4, simulations are taken to verify the effectiveness of the proposed method and analyze the space correlation function of the TSSRF and the resolution capability with the distance and the number of transmitters. Conclusions are drawn in Section 5.

## 2. The Proposed Bi-Static MSCI Method

The proposed method is expected to be applied for continuous observation of important areas in staring imaging geometry, which means that the radar is carried on a stationary/quasi-stationary platform. The bi-static MSCI system considered in this paper consists of two transmitting stations with two RRSs. By employing the bi-static radar system, it is anticipated to obtain a better TSSRF. In addition, both site-deploying and waveform design are considered in this paper.

**Figure 2:**
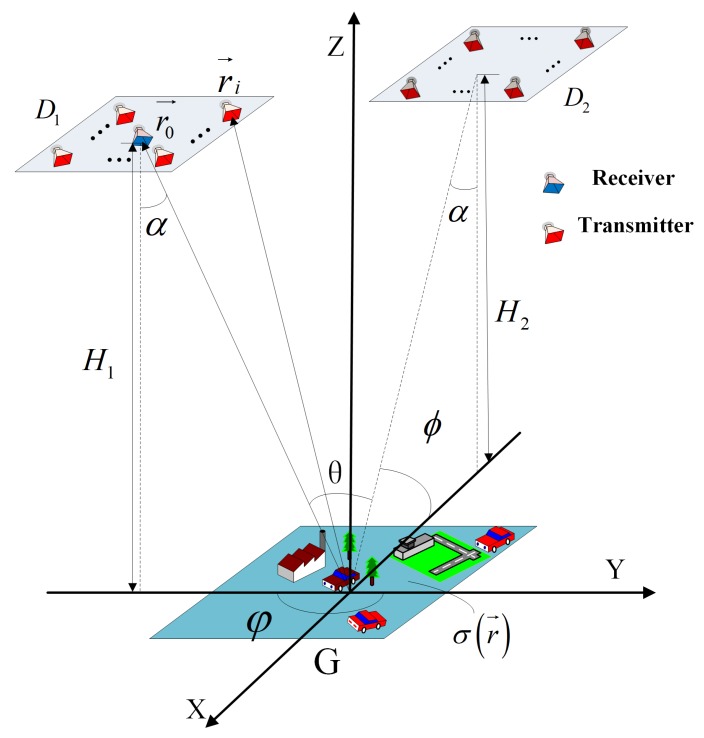
The proposed imaging geometry of bi-static MSCI.

Firstly, a new site-deploying scheme for bi-static MSCI is proposed, and its key point is that the two transmitting arrays observe the imaging area from two azimuth angles which differ by 90 degrees. The detailed imaging geometry is illustrated in Figure 2.

Let (x,y,z) be Cartesian coordinates with the origin *O* located at the center of the imaging area which is labeled *G*, and (r,φ,ϕ) denotes the according spherical coordinates, where azimuth angle φ denotes the angle between the orthogonal projection of the location vector on the XOY plane and the *Y* axis, and elevation angle ϕ denotes the angle between the location vector and the XOY plane. The plane XOY denotes the imaging plane and the *Z* axis is perpendicular to the ground.

The bi-static radar system is located in two stationary platforms above the ground, whose squint angles are both α, as shown in Figure 2. Each RRS is composed of a multiple antenna array labeled $D_{1}$ and $D_{2}$, respectively, and $D_{1}$ consists of $I_{1}$ antennas while $D_{2}$ consists of $I_{2}$ antennas. Meanwhile, the centers of the two transmit arrays are $\left(0,-H_{1}\tan\alpha ,H_{1}\right)$ and $\left(-H_{2}\tan\alpha ,0,H_{2}\right)$, respectively. Furthermore, the location vector of the *i*-th antenna is ${\vec{r}}_{i}$. In addition, the receive antenna is located at the center of $D_{1}$ with its location vector denoted as ${\vec{r}}_{0}=\left(0,-H_{1}\tan\alpha ,H_{1}\right)$. The viewing angle between the two stations to the imaging area is θ.

In practice, reflector geometry, shadowing, and the scattering characteristic caused by coherent scintillation can be strongly dependent on viewing angle, for this reason, θ is chosen to be less than $15^{\circ }$ [11].

**Figure 3:**
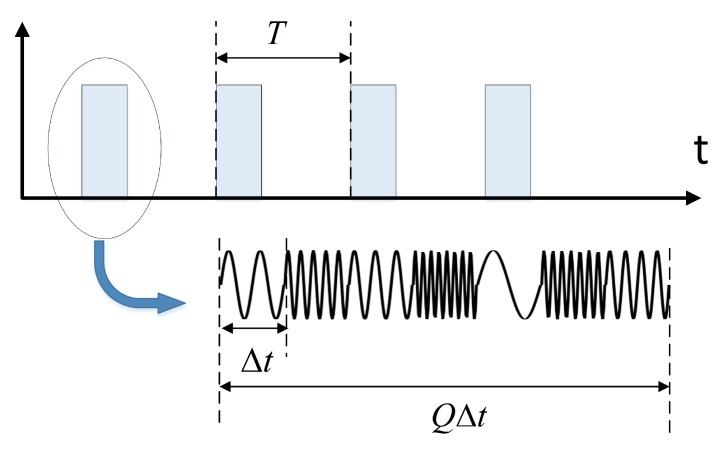
Waveform of the inner-and-inter pulse frequency hopping signals.

Secondly, for waveform design, this paper proposes to employ inner-and-inter pulse frequency hopping (IAIP-FH) signals and the waveform is illustrated in Figure 3. The transmitted signal of the *i*-th transmitter is

(1) $$S_{i}\left(t\right)=\sum_{l=1}^{L}\sum_{q=1}^{Q}u\left(t-q\Delta t-lT\right)e^{j2\pi f_{i,l,q}\left(t-lT\right)}$$

where *T* denotes the pulse period and Δt is the inner-pulse FH interval, *Q* is the number of FH code per pulse. Thus, the width of each pulse is QΔt, and u(t) is

$$u\left(t\right)\overset{\Delta }{\;=}\left\{\begin{array}{cc} 1 & 0<t<\Delta t\\ 0 & \mathrm{otherwise} \end{array}\right..$$

**Figure 4:**
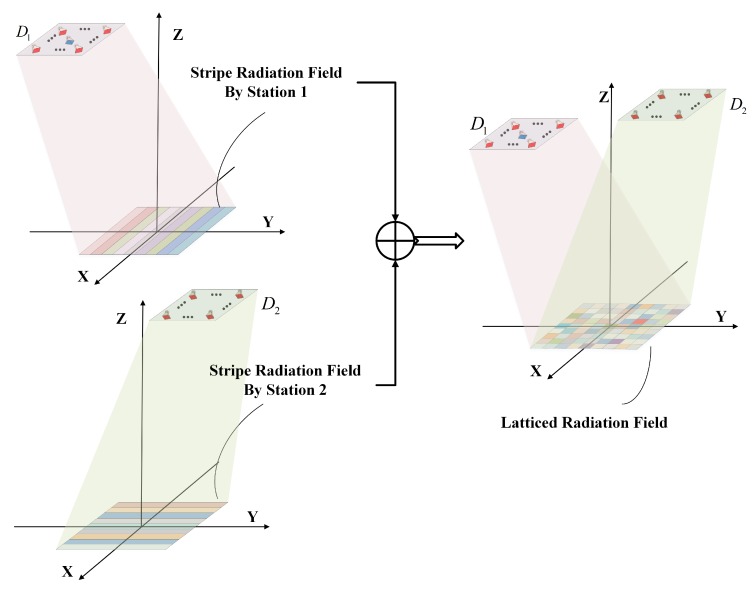
The superposition of the radiation field of bi-static MSCI.

By employing IAIP–FH signals, the radiation field of each RRS is a striped field with different distance stripes of the imaging area covered by different frequency stripes in one pulse. That is, the radiation field of $D_{1}$ forms *Q* frequency stripes perpendicular to the *Y* axis at the plane XOY and the radiation field of $D_{2}$ forms *Q* frequency stripes perpendicular to the X axis at the plane XOY, as illustrated in Figure 4.

The radiation field at the imaging plane is the superposition of the two “frequency stripe” fields and thus it forms the latticed radiation field and the field in the different lattice is independent, which is illustrated in Figure 4. Moreover, the grid size of the lattice is cΔtsinα×cΔtsinα, where *c* is the speed of light, Δt is the inner-pulse FH interval.

The radiation field at the location $\vec{r}$ can be expressed as [18,20]

(2) $$\begin{array}{cc} E(\vec{r},t)= & E_{rad1}\left(\vec{r},t\right)+E_{rad2}\left(\vec{r},t\right)\\ = & \sum_{i=1}^{I_{1}}\frac{A_{i}\left({\hat{\vec{R}}}_{i,\vec{r}}\right)}{4\pi |\vec{r}-{\vec{r}}_{i}|}\sum_{l=1}^{L}\sum_{q=1}^{Q}S_{i}\left(t-q\Delta t-lT-\tau_{i}\left(\vec{r}\right)\right)\\ + & \sum_{i=I_{1}+1}^{I_{1}+I_{2}}\frac{A_{i}\left({\hat{\vec{R}}}_{i,\vec{r}}\right)}{4\pi |\vec{r}-{\vec{r}}_{i}|}\sum_{l=1}^{L}\sum_{q=1}^{Q}S_{i}\left(t-q\Delta t-lT-\tau_{i}\left(\vec{r}\right)\right) \end{array}$$

where ${\hat{\vec{R}}}_{i,\vec{r}}=\left(\vec{r}-{\vec{r}}_{i}\right)/\left|\vec{r}-{\vec{r}}_{i}\right|$, $A_{i}\left(\cdot \right)$ denotes the radiation pattern of the *i*-th antenna, $\tau_{i}\left(\vec{r}\right)=\left|\vec{r}-{\vec{r}}_{i}\right|/c$, *c* is the speed of light, $S_{i}\left(t\right)$ is the transmitted signal of the *i*-th transmitter.

The radiation field interacts with the targets and the received echo signal can be expressed as [18,20]

(3) $$S_{echo}\left(t\right)=\int_{G}\sum_{i=1}^{I_{1}+I_{2}}\frac{A_{i}\left({\hat{\vec{R}}}_{i,\vec{r}}\right)A_{0}\left({\hat{\vec{R}}}_{0,\vec{r}}\right)}{{\left(4\pi \right)}^{2}\left|\vec{r}-{\vec{r}}_{i}\right|\left|\vec{r}-{\vec{r}}_{0}\right|}S_{i}\left(t-\tau_{i,0}\left(\vec{r}\right)\right)\sigma \left(\vec{r}\right)d\vec{r}+n\left(t\right)$$

where $\tau_{i,0}\left(\vec{r}\right)=\left(\left|\vec{r}-{\vec{r}}_{i}\right|+\left|\vec{r}-{\vec{r}}_{0}\right|\right)/c$, n(t) denotes the additive noise.

The modified radiation field is defined as [18]:

(4) $$\begin{array}{cc} E_{sca}\left(\vec{r},t\right)= & \sum_{i=1}^{I_{1}}\frac{A_{i}\left({\hat{\vec{R}}}_{i,\vec{r}}\right)A_{0}\left({\hat{\vec{R}}}_{0,\vec{r}}\right)}{{\left(4\pi \right)}^{2}\left|\vec{r}-{\vec{r}}_{i}\right|\left|\vec{r}-{\vec{r}}_{0}\right|}\sum_{l=1}^{L}\sum_{q=1}^{Q}S_{i}\left(t-q\Delta t-lT-\tau_{i,0}\left(\vec{r}\right)\right)\\ + & \sum_{i=I_{1}+1}^{I_{1}+I_{2}}\frac{A_{i}\left({\hat{\vec{R}}}_{i,\vec{r}}\right)A_{0}\left({\hat{\vec{R}}}_{0,\vec{r}}\right)}{{\left(4\pi \right)}^{2}\left|\vec{r}-{\vec{r}}_{i}\right|\left|\vec{r}-{\vec{r}}_{0}\right|}\sum_{l=1}^{L}\sum_{q=1}^{Q}S_{i}\left(t-q\Delta t-lT-\tau_{i,0}\left(\vec{r}\right)\right) \end{array}.$$

Therefore, the imaging equation of the integral form can be expressed as follows:

(5) $$S_{echo}\left(t\right)=\int_{G}E_{sca}\left(\vec{r},t\right)\sigma \left(\vec{r}\right)d\vec{r}+n\left(t\right).$$

In order to solve the imaging equation numerically, the imaging plane is discretized into N=U×K grid cells, with its size g×g, ${\vec{r}}_{j}$ denoting the center of the *j*-th grid cell. Let $\sigma_{j}=\sigma \left({\vec{r}}_{j}\right)$ denotes the backscattering coefficient of the *j*-th grid cell. In addition, the echo signal is also sampled, thus the final imaging equation is

(6) $$S_{echo}=E_{sca}\cdot \sigma +n$$

where $S_{echo}={\left[S_{echo}\left(t_{1}\right),S_{echo}\left(t_{2}\right),\cdots ,S_{echo}\left(t_{M}\right)\right]}^{T}\in C^{M\times 1}$, $\sigma ={\left[\sigma_{1},\sigma_{2},\cdots ,\sigma_{N}\right]}^{T}\in C^{N\times 1}$, $n\in C^{M\times 1}$ is the additive noise vector, $E_{sca}\in C^{M\times N}$ is the radiation field matrix with ${\left[E_{sca}\right]}_{kj}=E_{sca}\left({\vec{r}}_{j},t_{k}\right)$.

The reconstruction for σ using $E_{sca}$ and $S_{echo}$ can be expressed as

$$\sigma =℘[S_{echo},E_{sca}]$$

where, *℘* denotes the operator of the correlated process (CP) algorithms. The common CP algorithms include, for example, the first order CP algorithm [18], the Tikhonov regularization method [17,18], and some sparse recovery methods, such as orthogonal matching pursuit (OMP) [27,28], sparse Bayes learning (SBL) [29,30] algorithm, and so on. Recently, some structured compressive recovery algorithms [31] have been applied into MSCI due to their ability of exploiting the elaborate structure information of the targets [32,33,34]. In radar imaging applications, the targets typically have cluster structures, thus the cluster prior of the targets is considered to develop the algorithms in References [32,33,34].

**Figure 5:**
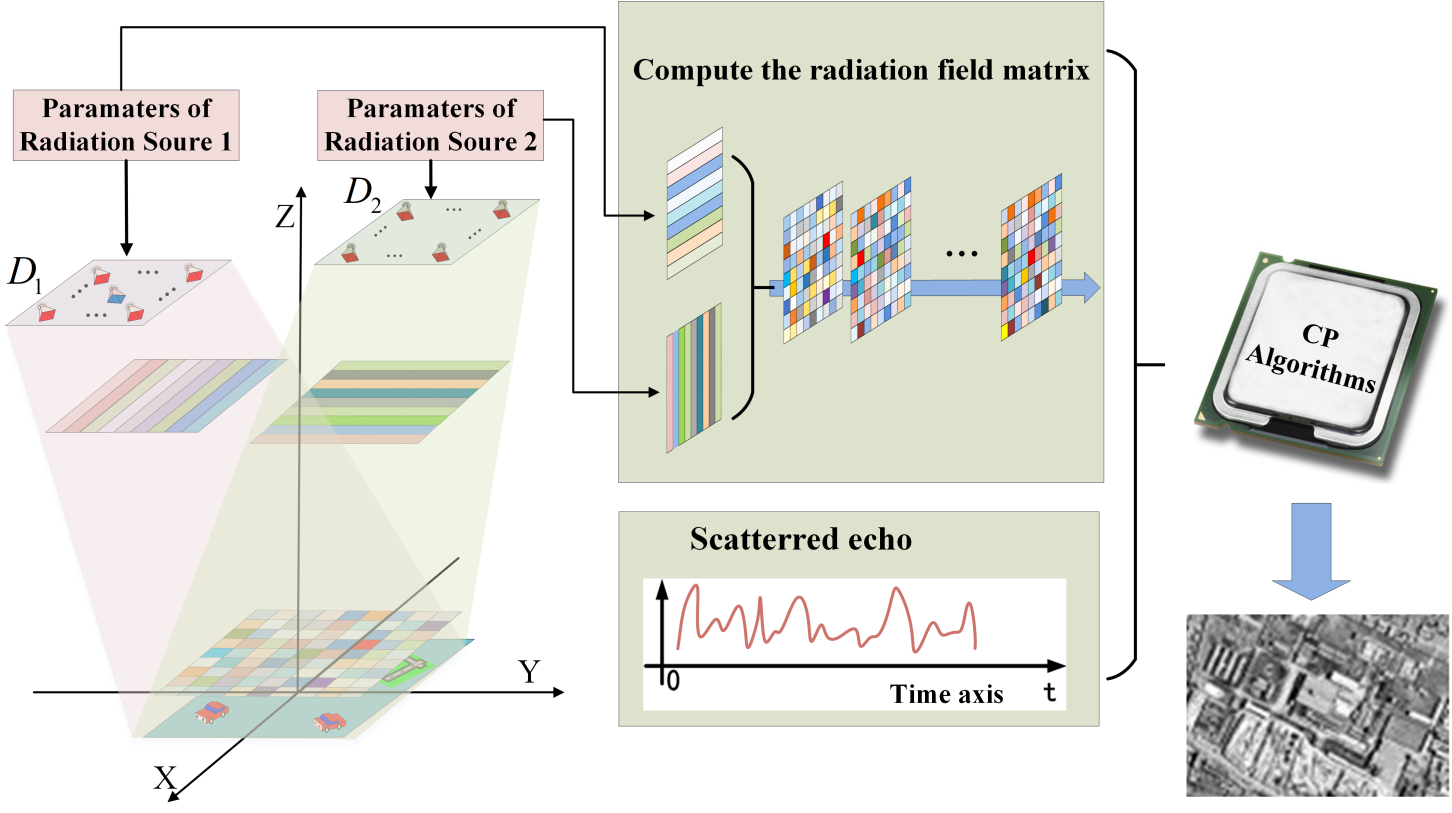
Imaging geometry and procedure of the proposed method.

The detailed procedure of the proposed method is shown in Figure 5.

**Figure 6:**
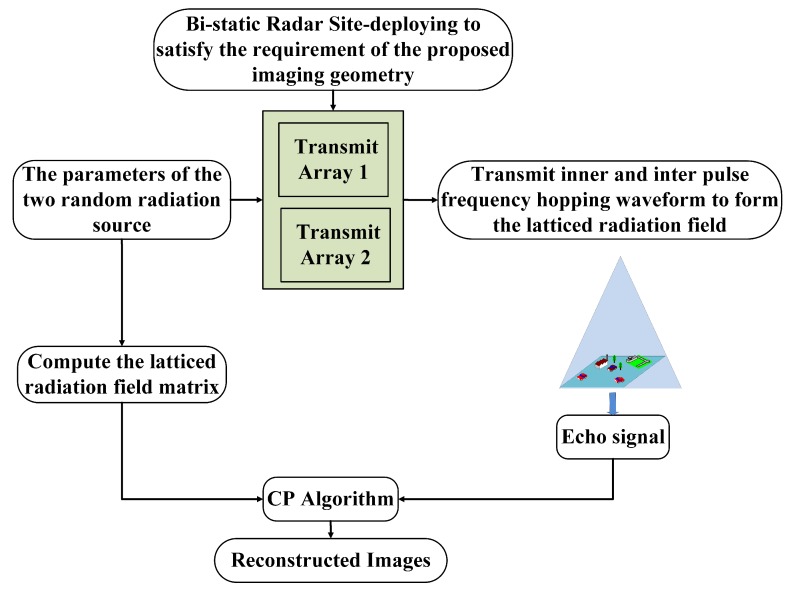
Flow chart of the proposed bi-static MSCI method.

To sum up, the detailed imaging procedure of the proposed method is as follows: (1) perform site-deploying to satisfy the requirements of the proposed imaging geometry; (2) the two RRSs synchronously transmit the IAIP-FH signals to construct a latticed field at the imaging plane; (3) sample the received signal and compute the modified radiation field matrix based on the waveform parameters; (4) correlation process (CP) of the sampled signal and the matrix to obtain the image. The imaging procedure flow of the proposed method is illustrated in Figure 6.

In addition, the transmitters at the two stations should be accurately synchronized in our proposed method. Typically, the world wide accessible GPS signals and the microwave link established between the two stations can realize high-precision time synchronization [35]. Nevertheless, synchronization errors cannot be fully eliminated. Readers who are interested in calibration methods related to synchronization errors in MSCI can refer to Reference [36].

## 3. Analysis of the Space Correlation Function of the Proposed Method

According to the foregoing, the proposed method constructs a latticed radiation field, thus the stochastic characteristic of the radiation field is greatly improved. Commonly, the space correlation function is used to measure the stochastic characteristic of the radiation field [15,16], which has been pointed out to be related with the imaging system’s point spread function (PSF) in Reference [37]. In this subsection, the space correlation function is analyzed.

The space correlation function of $E(\vec{r},t)$ at two different locations ${\vec{r}}_{j}$ and ${\vec{r}}_{j}+\Delta \vec{r}$ is defined as follows [15,16]:

(7) $$\begin{array}{cc} R({\vec{r}}_{j},\Delta \vec{r}) & =\langle E_{sca}\left({\vec{r}}_{j},t\right),E_{sca}\left({\vec{r}}_{j}+\Delta \vec{r},t\right)\rangle \\ & =\frac{1}{M}\sum_{k=1}^{M}E_{sca}\left({\vec{r}}_{j},t_{k}\right),E_{sca}\left({\vec{r}}_{j}+\Delta \vec{r},t_{k}\right) \end{array}$$

where ${\vec{r}}_{j}=\left(x_{j},y_{j},0\right)$ and $\Delta \vec{r}=\left(\Delta x,\Delta y,0\right)$.

By substituting (4) into (7), $R({\vec{r}}_{j},\Delta \vec{r})$ can be expressed in (8).

(8) $$\begin{array}{c} R\left({\vec{r}}_{j},\Delta \vec{r}\right)=C_{0}\sum_{i=1}^{I}\frac{A_{i}\left({\hat{\vec{R}}}_{i,{\vec{r}}_{j}}\right)A_{0}\left({\hat{\vec{R}}}_{0,{\vec{r}}_{j}}\right)A_{i}\left({\hat{\vec{R}}}_{i,{\vec{r}}_{j}+\Delta \vec{r}}\right)A_{0}\left({\hat{\vec{R}}}_{0,{\vec{r}}_{j}+\Delta \vec{r}}\right)}{\left|{\vec{r}}_{j}-\vec{r_{i}}\right|\left|{\vec{r}}_{j}-\vec{r_{0}}\right|\left|{\vec{r}}_{j}+\Delta \vec{r}-\vec{r_{i}}\right|\left|{\vec{r}}_{j}+\Delta \vec{r}-\vec{r_{0}}\right|}S_{i}\left(t_{k}-\tau_{i,0}\left({\vec{r}}_{j}\right)\right)S_{i}\left(t_{k}-\tau_{i,0}\left({\vec{r}}_{j}+\Delta \vec{r}\right)\right)\\ +C_{0}\sum_{i_{1}\neq i_{2}}^{I}\frac{A_{i_{1}}\left({\hat{\vec{R}}}_{i_{1},{\vec{r}}_{j}}\right)A_{0}\left({\hat{\vec{R}}}_{0,{\vec{r}}_{j}}\right)A_{i_{2}}\left({\hat{\vec{R}}}_{i_{2},{\vec{r}}_{j}+\Delta \vec{r}}\right)A_{0}\left({\hat{\vec{R}}}_{0,{\vec{r}}_{j}+\Delta \vec{r}}\right)}{\left|{\vec{r}}_{j}-\vec{r_{i_{1}}}\right|\left|{\vec{r}}_{j}-\vec{r_{0}}\right|\left|{\vec{r}}_{j}+\Delta \vec{r}-\vec{r_{i_{2}}}\right|\left|{\vec{r}}_{j}+\Delta \vec{r}-\vec{r_{0}}\right|}\sum_{k=1}^{M}S_{i_{1}}\left(t_{k}-\tau_{i_{1},0}\left({\vec{r}}_{j}\right)\right)S_{i_{2}}\left(t_{k}-\tau_{i_{2},0}\left({\vec{r}}_{j}+\Delta \vec{r}\right)\right)\\ \approx C\sum_{i=1}^{I}\sum_{k=1}^{M}S_{i}\left(t_{k}-\tau_{i,0}\left({\vec{r}}_{j}\right)\right)S_{i}\left(t_{k}-\tau_{i,0}\left({\vec{r}}_{j}+\Delta \vec{r}\right)\right)+C\sum_{i_{1}\neq i_{2}}^{I}\sum_{k=1}^{M}S_{i_{1}}\left(t_{k}-\tau_{i_{1},0}\left({\vec{r}}_{j}\right)\right)S_{i_{2}}\left(t_{k}-\tau_{i_{2},0}\left({\vec{r}}_{j}+\Delta \vec{r}\right)\right) \end{array}$$

where $C_{0}=\frac{1}{{\left(4\pi \right)}^{4}M}$.

In (8), the heights of the two stations are supposed to be the same, so the imaging distances between the imaging region and the two station are the same. Under far field condition $|\vec{r_{j}}|\gg |\Delta \vec{r}|$, we can make approximations that $\frac{1}{|{\vec{r}}_{j}-{\vec{r}}_{0}|}\approx \frac{1}{|{\vec{r}}_{j}-{\vec{r}}_{i}\left|\right|}\approx \frac{1}{|{\vec{r}}_{j}+\Delta \vec{r}-{\vec{r}}_{0}|}\approx \frac{1}{|{\vec{r}}_{j}+\Delta \vec{r}-{\vec{r}}_{i}|}\approx \frac{1}{z}$, where *z* is the imaging distance. Moreover, ${\vec{r}}_{j}$ and ${\vec{r}}_{j}+\Delta \vec{r}$ are close to each other within the beam coverage. In addition, the radiation patterns of all the antennas are supposed to have the same characteristics. Thus, $A_{i}\left({\hat{\vec{R}}}_{i,{\vec{r}}_{j}}\right)\approx A_{i}\left({\hat{\vec{R}}}_{i,{\vec{r}}_{j}+\Delta \vec{r}}\right)$, $A_{0}\left({\hat{\vec{R}}}_{i,{\vec{r}}_{j}}\right)\approx A_{0}\left({\hat{\vec{R}}}_{i,{\vec{r}}_{j}+\Delta \vec{r}}\right)$, $\frac{1}{{\left(4\pi \right)}^{4}M}\frac{A_{i}\left({\hat{\vec{R}}}_{i,{\vec{r}}_{j}}\right)A_{0}\left({\hat{\vec{R}}}_{0,{\vec{r}}_{j}}\right)A_{i}\left({\hat{\vec{R}}}_{i,{\vec{r}}_{j}+\Delta \vec{r}}\right)A_{0}\left({\hat{\vec{R}}}_{0,{\vec{r}}_{j}+\Delta \vec{r}}\right)}{\left|{\vec{r}}_{j}-\vec{r_{i}}\right|\left|{\vec{r}}_{j}-\vec{r_{0}}\right|\left|{\vec{r}}_{j}+\Delta \vec{r}-\vec{r_{i}}\right|\left|{\vec{r}}_{j}+\Delta \vec{r}-\vec{r_{0}}\right|}\approx C$ is almost a constant. The first term on the right of (8) is the cross-correlation of the different transmitters’ signals and the second term is the self-correlation. Since the frequency codes of each transmitter are randomly and independently selected, thus the cross-correlation of the different transmitters is much less than the self-correlation, thus the first term is neglected hereinafter:

(9) $$\begin{array}{cc} R({\vec{r}}_{j},\Delta \vec{r})\approx & \sum_{l=1}^{L}\sum_{i=1}^{I}u\left(t-q\Delta t-lT-\tau_{i,0}\left({\vec{r}}_{j}+\Delta \vec{r}\right)\right)\sum_{k=1}^{M}e^{j2\pi f_{i,j,q}\left(\tau_{i,0}\left({\vec{r}}_{j}+\Delta \vec{r}\right)-\tau_{i,0}\left({\vec{r}}_{j}\right)\right)}\\ = & \sum_{l=1}^{L}\sum_{i=1}^{I}\sum_{k=1}^{M}e^{j2\pi f_{i,j,q}\left(\tau_{i,0}\left({\vec{r}}_{j}+\Delta \vec{r}\right)-\tau_{i,0}\left({\vec{r}}_{j}\right)\right)}. \end{array}$$

Notice that the constant *C* is omitted here. Furthermore, $f_{i,j,q}\in \left[f_{L},f_{H}\right]$, where $f_{L}$, $f_{H}$ are the lower and upper bound of the transmitted frequency band. In the proposed IAIP-FH signals, $f_{i,j,q}$ is uniformly and randomly selected in $\left[f_{L},f_{H}\right]$, so

(10) $$\langle e^{j2\pi f_{i,j,q}\left(\tau_{i,0}\left({\vec{r}}_{j}+\Delta \vec{r}\right)-\tau_{i,0}\left({\vec{r}}_{j}\right)\right)}\rangle \approx B\sum_{i}^{I}e^{j2\pi f_{c}\left(\tau_{i,0}\left({\vec{r}}_{j}+\Delta \vec{r}\right)-\tau_{i,0}\left({\vec{r}}_{j}\right)\right)}\times sinc\left(B\left(\tau_{i,0}\left({\vec{r}}_{j}+\Delta \vec{r}\right)-\tau_{i,0}\left({\vec{r}}_{j}\right)\right)\right)$$

where 〈·〉 denotes expectation and $sinc\left(x\right)=\frac{\sin(\pi x)}{\pi x}$, $f_{c}=\left(f_{L}+f_{H}\right)/2$.

Therefore, the space correlation function $R({\vec{r}}_{j},\Delta \vec{r})$ can be expressed as

(11) $$R\left({\vec{r}}_{j},\Delta \vec{r}\right)\approx R_{1}\left({\vec{r}}_{j},\Delta \vec{r}\right)+R_{2}\left({\vec{r}}_{j},\Delta \vec{r}\right)$$

where

(12) $$\begin{array}{cc} R_{1}\left({\vec{r}}_{j},\Delta \vec{r}\right)= & B\sum_{i=1}^{I_{1}}e^{j2\pi f_{c}\left(\tau_{i,0}\left({\vec{r}}_{j}+\Delta \vec{r}\right)-\tau_{i,0}\left({\vec{r}}_{j}\right)\right)}sinc\left(B\left(\tau_{i,0}\left({\vec{r}}_{j}+\Delta \vec{r}\right)-\tau_{i,0}\left({\vec{r}}_{j}\right)\right)\right)\\ R_{2}\left({\vec{r}}_{j},\Delta \vec{r}\right)= & B\sum_{i=I_{1}+1}^{I_{1}+I_{2}}e^{j2\pi f_{c}\left(\tau_{i,0}\left({\vec{r}}_{j}+\Delta \vec{r}\right)-\tau_{i,0}\left({\vec{r}}_{j}\right)\right)}sinc\left(B\left(\tau_{i,0}\left({\vec{r}}_{j}+\Delta \vec{r}\right)-\tau_{i,0}\left({\vec{r}}_{j}\right)\right)\right). \end{array}$$

In (12), $\tau_{i,0}\left({\vec{r}}_{j}\right)=\left|{\vec{r}}_{j}-{\vec{r}}_{i}\right|\left|{\vec{r}}_{j}-{\vec{r}}_{0}\right|/c$, by substituting location vector ${\vec{r}}_{i}=\left(\left|{\vec{r}}_{i}\right|,\varphi_{i},\phi_{i}\right)$ of the *i*-th antenna and the location vector ${\vec{r}}_{0}=\left(\left|{\vec{r}}_{0}\right|,\varphi_{0},\phi_{0}\right)=\left(H_{1}/\tan\alpha ,\pi ,\frac{\pi }{2}-\alpha \right)$ of the receive antenna into $\tau_{i,0}\left({\vec{r}}_{j}\right)$, and applying far field approximation:

(13) $$\begin{array}{cc} c\tau_{i,0}\left({\vec{r}}_{j}\right)\approx & |{\vec{r}}_{i}\left|+\right|{\vec{r}}_{0}|-x_{j}\left(\cos\phi_{i}\sin\varphi_{i}+\cos\phi_{0}\sin\varphi_{0}\right)-y_{j}\left(\cos\phi_{i}\cos\varphi_{i}+\cos\phi_{0}\cos\varphi_{0}\right)\\ = & |{\vec{r}}_{i}\left|+\right|{\vec{r}}_{0}|-x_{j}\cos\phi_{i}\sin\varphi_{i}-y_{j}\left(\cos\phi_{i}\cos\varphi_{i}-\cos\phi_{0}\right). \end{array}$$

Substitute (13) into $c(\tau_{i}\left({\vec{r}}_{j}+\Delta \vec{r}\right)-\tau_{i}\left({\vec{r}}_{j}\right))$:

(14) $$c\left(\tau_{i,0}\left({\vec{r}}_{j}+\Delta \vec{r}\right)-\tau_{i,0}\left({\vec{r}}_{j}\right)\right)=-\Delta x\cos\phi_{i}\sin\varphi_{i}-\Delta y\left(\cos\phi_{i}\cos\varphi_{i}-\cos\phi_{0}\right).$$

The receive antenna is at the center of $D_{1}$ as illustrated in Figure 1, its azimuth angle $\varphi_{0}$ is π . Additionally, the azimuth angle of the other antennas in $D_{1}$ is approximately equal to π, and their elevation angle $\phi_{i}$ is approximately equal to $\phi_{0}$. Thus, we can make an approximation that $\sin\varphi_{i}=-\sin\left(\varphi_{0}-\pi \right)\approx \pi -\phi_{i}$, $\cos\varphi_{i}\approx -1$ and substitute these into $R_{1}\left({\vec{r}}_{j},\Delta \vec{r}\right)$:

(15) $$\begin{array}{cc} R_{1}\left({\vec{r}}_{j},\Delta \vec{r}\right) & =B\sum_{i=1}^{I_{1}}e^{j2\pi f_{c}\left(\tau_{i,0}\left({\vec{r}}_{j}+\Delta \vec{r}\right)-\tau_{i,0}\left({\vec{r}}_{j}\right)\right)}sinc\left(\frac{B}{c}\left(\Delta x\cos\phi_{i}\left(\pi -\phi_{i}\right)-\Delta y\left(\cos\phi_{i}+\cos\phi_{0}\right)\right)\right)\\ & \approx B\sum_{i=1}^{I_{1}}e^{j2\pi f_{c}\left(\tau_{i,0}\left({\vec{r}}_{j}+\Delta \vec{r}\right)-\tau_{i,0}\left({\vec{r}}_{j}\right)\right)}sinc\left(\frac{B}{c}\left(\Delta x\left(\pi -\phi_{i}\right)\cos\phi_{i}-2\Delta y\cos\phi_{0}\right)\right). \end{array}$$

**Figure 7:**
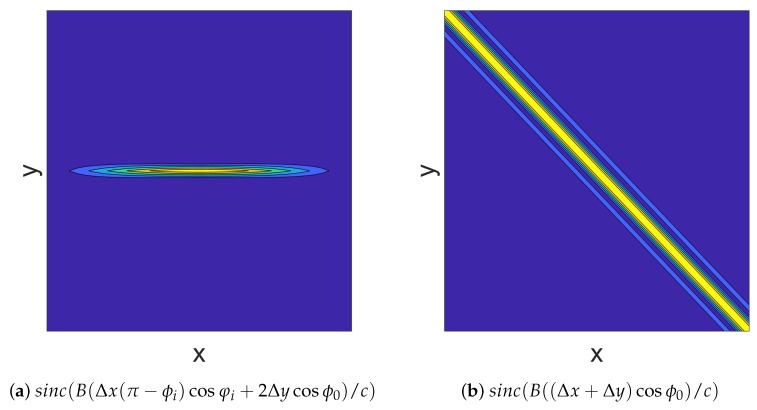
The contour map of two-dimensional sinc function.

On the basis of (15), the two-dimensional function $sinc(\frac{B}{c}\left(\Delta x\cos\varphi_{i}\sin\phi_{i}-2\Delta y\cos\phi_{0}\right))$ determines the envelop of $R_{1}\left({\vec{r}}_{j},\Delta \vec{r}\right)$ and its contour map is illustrated in Figure 7a. In addition, its 3 dB beam width along *X* axis and *Y* axis can be easily computed, $Beam1_{3dB}^{X}=0.882c/B\cos\phi_{0}\left|\pi -\varphi_{i}\right|$, $Beam1_{3dB}^{Y}=0.441c/B\cos\phi_{0}$. Since $Beam1_{3dB}^{X}/Beam1_{3dB}^{Y}\approx \frac{2}{|\pi -\varphi_{i}|}\gg 1$, thus the 3 dB beam width along *X* axis of $R_{1}\left({\vec{r}}_{j},\Delta \vec{r}\right)$ is far wider than that along *Y* axis.

For the antennas of $D_{2}$, their azimuth angles $\varphi_{i}$ are approximately equal to $\frac{3\pi }{2}$, thus $|\varphi_{i}-\frac{3\pi }{2}|\ll 1$, so $\sin\varphi_{i}\approx -1$, $\cos\varphi_{i}\approx 0$. Their elevation angle is approximately equal to $\phi_{0}$.

Thus, $R_{2}\left({\vec{r}}_{j},\Delta \vec{r}\right)$ is

(16) $$\begin{array}{cc} R_{2}\left({\vec{r}}_{j},\Delta \vec{r}\right) & =B\sum_{i=I_{1}+1}^{I_{1}+I_{2}}e^{j2\pi f_{c}\left(\tau_{i,0}\left({\vec{r}}_{j}+\Delta \vec{r}\right)-\tau_{i,0}\left({\vec{r}}_{j}\right)\right)}\times sinc\left(\frac{B}{c}\left(\Delta x\cos\phi_{i}\sin\varphi_{i}+\Delta y\left(\cos\phi_{i}\cos\varphi_{i}-\cos\phi_{0}\right)\right)\right)\\ & \approx B\sum_{i=I_{1}+1}^{I_{1}+I_{2}}e^{j2\pi f_{c}\left(\tau_{i,0}\left({\vec{r}}_{j}+\Delta \vec{r}\right)-\tau_{i,0}\left({\vec{r}}_{j}\right)\right)}\times sinc\left(\frac{B}{c}\left(\left(\Delta x+\Delta y\right)\cos\phi_{0}\right)\right). \end{array}$$

The contour map of the function $sinc(\frac{B}{c}\left(\left(\Delta x+\Delta y\right)\cos\phi_{0}\right))$ is illustrated in Figure 7b. It can be easily seen that when Δx=−Δy, that is, $\Delta \vec{r}$ moves along vector (1,−1,0), the value of $R_{2}\left({\vec{r}}_{j},\Delta \vec{r}\right)$ stays the same, so the 3 dB beam width of $R_{2}\left({\vec{r}}_{j},\Delta \vec{r}\right)$ along vector (1,−1,0) is wide. On the contrary, when $\Delta \vec{r}$ moves along vector (1,1,0), the value of $R_{2}\left({\vec{r}}_{j},\Delta \vec{r}\right)$ decreases fast, so the 3 dB beam width of $R_{2}\left({\vec{r}}_{j},\Delta \vec{r}\right)$ along vector (1,1,0) is sharp and its 3 dB beam width is $0.882c/B\sqrt{2}\cos\varphi_{0}$.

By Substituting (15) and (16) into (11), $R({\vec{r}}_{j},\Delta \vec{r})$ can be expressed as

(17) $$\begin{array}{cc} R({\vec{r}}_{j},\Delta \vec{r})= & B\sum_{i=1}^{I_{1}}e^{j2\pi f_{c}\left(\tau_{i,0}\left({\vec{r}}_{j}+\Delta \vec{r}\right)-\tau_{i,0}\left({\vec{r}}_{j}\right)\right)}sinc\left(\frac{B}{c}\left(\Delta x\left(\pi -\phi_{i}\right)\cos\varphi_{i}+2\Delta y\cos\phi_{0}\right)\right)\\ & +B\sum_{i=I_{1}+1}^{I_{1}+I_{2}}e^{j2\pi f_{c}\left(\tau_{i,0}\left({\vec{r}}_{j}+\Delta \vec{r}\right)-\tau_{i,0}\left({\vec{r}}_{j}\right)\right)}sinc\left(\frac{B}{c}\left(\left(\Delta x+\Delta y\right)\cos\phi_{0}\right)\right). \end{array}$$

Thus,

(18) $$\left|\frac{R({\vec{r}}_{j},\Delta \vec{r})}{R({\vec{r}}_{j},0)}\right|=\frac{\sum_{i=I_{1}+1}^{I_{1}+I_{2}}sinc\left(\frac{B}{c}\left(\left(\Delta x+\Delta y\right)\cos\phi_{0}\right)\right)}{I_{1}+I_{2}}+\frac{\sum_{i=1}^{I_{1}}sinc\left(\frac{B}{c}\left(\Delta x\left(\pi -\phi_{i}\right)\cos\varphi_{i}+2\Delta y\cos\phi_{0}\right)\right)}{I_{1}+I_{2}}.$$

On the basis of (18), for each location ${\vec{r}}_{j}$ in the imaging area, $\Delta \vec{r}$ moves along any direction for $\left|\Delta x\right|\geq \frac{c}{B\cos\phi_{0}}$ or $\left|\Delta y\right|\geq \frac{c}{B\cos\phi_{0}}$, one can get that $\left|\frac{R({\vec{r}}_{j},\Delta \vec{r})}{R({\vec{r}}_{j},0)}\right|\leq 0.6<\frac{\sqrt{2}}{2}$. That means the 3 dB beam width of $R({\vec{r}}_{j},\Delta \vec{r})$ along any direction is less than $\frac{c}{B\cos\phi_{0}}$, which does not change with the imaging distance. On the other hand, the side lobe of the $R_{1}\left({\vec{r}}_{j},\Delta \vec{r}\right)$ and $R_{2}\left({\vec{r}}_{j},\Delta \vec{r}\right)$ are superposed, thus along the *X* axis and the vector (1,−1,0), $R({\vec{r}}_{j},\Delta \vec{r})$ has high side lobes. For example, when $\Delta \vec{r}=\left(\Delta x,0,0\right)$, and $\frac{c}{B\cos\phi_{0}}\leq \left|\Delta x\right|\leq \frac{c}{10\left(\pi -\phi_{i}\right)B\cos\phi_{0}}$, it is always found that $\left|\frac{R({\vec{r}}_{j},\Delta \vec{r})}{R({\vec{r}}_{j},0)}\right|\geq \frac{\sum_{i=1}^{I_{1}}sinc\left(-\Delta x\left(\pi -\phi_{i}\right)\pi B/c\right)}{I_{1}+I_{2}}\geq 0.98\frac{I_{1}}{I_{2}}=0.49$, which means the side lobe is high.

For the mono-static MSCI, the space correlation function is the same as when all the transmitters are placed in $D_{1}$, so it can be expressed as

(19) $$R\left({\vec{r}}_{j},\Delta \vec{r}\right)=B\sum_{i=1}^{I_{1}+I_{2}}e^{j2\pi f_{c}\left(\tau_{i,0}\left({\vec{r}}_{j}+\Delta \vec{r}\right)-\tau_{i,0}\left({\vec{r}}_{j}\right)\right)}sinc\left(\frac{B}{c}\left(\Delta x\left(\pi -\phi_{i}\right)\cos\varphi_{i}+2\Delta y\cos\phi_{0}\right)\right).$$

Therefore, the 3 dB beam width along the *X* axis of the space correlation function $R({\vec{r}}_{j},\Delta \vec{r})$ of mono-static radar is far wider than that along the *Y* axis.

In the following paragraphs, the choice of different azimuth angles of the two stations that differ by 90 degrees is discussed. Suppose the location of Station 1 stays the same, while the location of the center of Station 2 is changed to $\left(H\tan\alpha \sin\varphi_{2},H\tan\alpha \cos\varphi_{2},H\right)$. Therefore, after similar derivations, $R({\vec{r}}_{j},\Delta \vec{r})$ can be expressed as

(20) $$\begin{array}{c} \left|\frac{R({\vec{r}}_{j},\Delta \vec{r})}{R({\vec{r}}_{j},0)}\right|=\frac{\sum_{i=1}^{I_{1}}sinc\left(\frac{B}{c}\left(\Delta x\left(\pi -\phi_{i}\right)\cos\varphi_{i}+2\Delta y\cos\phi_{0}\right)\right)}{I_{1}+I_{2}}\\ +\frac{\sum_{i=I_{1}+1}^{I_{1}+I_{2}}sinc\left(\frac{B}{c}\left(\Delta x\cos\phi_{0}\left(\sin\varphi_{2}+\left(\varphi_{i}-\varphi_{2}\right)\cos\varphi_{2}\right)+\Delta y\cos\phi_{0}\left(\cos\varphi_{2}-\left(\varphi_{i}-\varphi_{2}\right)\sin\varphi_{2}-1\right)\right)\right)}{I_{1}+I_{2}} \end{array}.$$

Since $\varphi_{i}-\varphi_{2}\ll 1$, the 3 dB width of $R({\vec{r}}_{j},\Delta \vec{r})$ along the Y axis and the vector $\left(\cos\phi_{0}\sin\varphi_{2},\cos\phi_{0}\left(\cos\varphi_{2}-1\right),0\right)$ is sharp and the values of these 3 dB width are $0.441c/B\cos\phi_{0}$ and $0.882c/B\cos\phi_{0}\sqrt{2(1-\cos\varphi_{2})}$, respectively. In addition, as mentioned in Section 2, θ is limited and there is a condition based on the imaging geometry such that $\sin\alpha =\frac{1-\cos\theta }{1+\cos\varphi_{2}}$. Since $\cos\phi_{0}=\sin\alpha$, one gets that $0.441c/B\cos\phi_{0}=0.441c/B\sin\alpha$ and $0.882c/B\cos\phi_{0}\sqrt{2(1-\cos\varphi_{2})}=\frac{0.441\sqrt{2}c}{B\sqrt{\left(1-\cos\theta \right)}}\sqrt{\frac{1+\cos\varphi_{2}}{1-\cos\varphi_{2}}}$.

To sum up, on the one hand, the 3 dB beam width of $R({\vec{r}}_{j},\Delta \vec{r})$ along the vector $\left(\cos\phi_{0}\sin\varphi_{2},\cos\phi_{0}\left(\cos\varphi_{2}-1\right),0\right)$ and the Y axis are anticipated to be sharp, on the other hand, the angle between the side lobes is anticipated to be as large as possible. Therefore, the choice of $\varphi_{2}$ is a compromise of the 3 dB width of $R({\vec{r}}_{j},\Delta \vec{r})$ and the side lobes’ directions.

In conclusion, choosing different azimuth angles of the two stations that differ by 90 degrees is a reasonable and comprehensive consideration.

## 4. Simulations

The 3 dB beam width of the space correlation function of the proposed method does not change with the imaging distance, and the stochastic characteristic of the radiation field is greatly improved. To verify the effectiveness of the proposed method and analyze its resolution capabilities, five groups of numerical simulations are presented in this section. First, simulations are presented to compare the imaging results of mono-static and bi-static MSCI. Then, the space correlation functions of mono-static MSCI and the proposed method are compared and the space correlation functions of different distances are illustrated to verify the derivation of Section 3. Finally, the resolution capability with the number of transmitters and the imaging distance is analyzed using simulations.

The radar system used in the simulations in this section works at X band frequency. In addition, the antennas of the bi-static and mono-static MSCI are all equally placed in the transmitting array. Furthermore, the mono-static MSCI system adopts a 1.6 m × 3.2 m array, while the bi-static MSCI system adopts two 1.6 m × 1.6 m arrays. Some parameters are given in Table 1. The parameters related to the imaging distance, pulse width, and inner-pulse FH interval are different in each different simulation.

### 4.1. Imaging Simulations for Mono-Static and Bi-Static MSCI

In this subsection, simulations are presented to compare the imaging results of mono-static and bi-static MSCI with different imaging distance. To clearly describe the imaging performance, the normalized mean square error (NMSE) is used to quantify the reconstruction effect of the target imaging; the definition of which is $NMSE_{dB}=20\mathrm{lg}\left[{∥\hat{\sigma }-\sigma ∥}_{2}/{∥\sigma ∥}_{2}\right]$, where σ denotes the target image and $\hat{\sigma }$ denotes the reconstruction image.

The system parameters, including the pulse width, number of transmitters, and inner-pulse FH interval, are given in Table 2, and the other parameters are the same as those in Table 1. The height of the radar platform varies from 500 m to 10 km, and the according imaging region size varies from 20 m × 20 m to 160 m × 160 m . The signal to noise ratio (SNR) is set to 25 dB in the five simulations. The PC-SBL algorithm exploits the cluster structure prior to the targets, thus it has a better and more stable performance than other CP algorithms in mostly situations [31,34]. Besides, the target images in the simulations indeed have cluster structures. Therefore, the PC-SBL algorithm is adopted in the following simulations.

**Figure 8:**
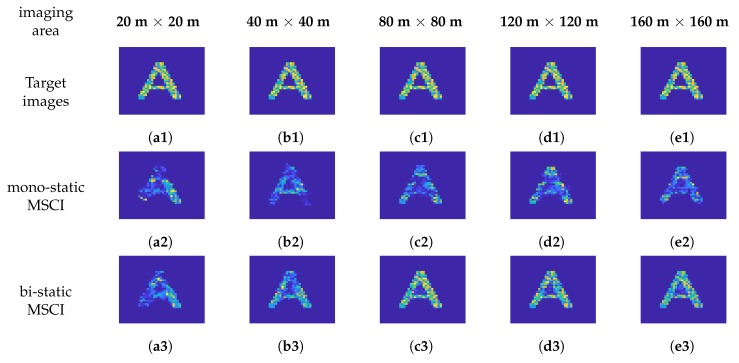
The imaging results of mono-static and bi-static MSCI. (**a**1–**e**1) the target images when the heights of the radar platform are 500 m, 1 km, 2 km, 5 km, and 10 km, respectively; (**a**2–**e**2) the reconstructed images using mono-static MSCI when the heights of the radar platform are 500 m, 1 km, 2 km, 5 km, and 10 km, respectively; (**a**3–**e**3) the reconstructed images by bi-static MSCI when the heights of the radar platform are 500 m, 1 km, 2 km, 5 km, and 10 km, respectively.

The imaging results of mono-static MSCI and the proposed bi-static MSCI with different imaging distances are illustrated in Figure 8. It can be seen that the target images (a3, b3, c3, d3, e3) reconstructed by bi-static MSCI have clear outlines and are easily identified, while the outlines of the target images (a2, b2, c2, d2, e2) reconstructed by mono-static MSCI are distorted and the targets cannot even be identified. The NMSEs of the reconstructed images given in Table 3 are averaged by five Monte Carlo trials for each imaging distance. It can be seen that the NMSEs of bi-static MSCI are lower which means better results. In brief, the bi-static MSCI can acquire clearer images and better results than the mono-static MSCI, which shows the effectiveness of the proposed method.

### 4.2. The Space Correlation Functions of Mono-Static and Bi-Static MSCI

In Section 3, the space correlation functions of mono-static and bi-static MSCI are analyzed. To verify the formula derivation, the following simulation is presented.

**Figure 9:**
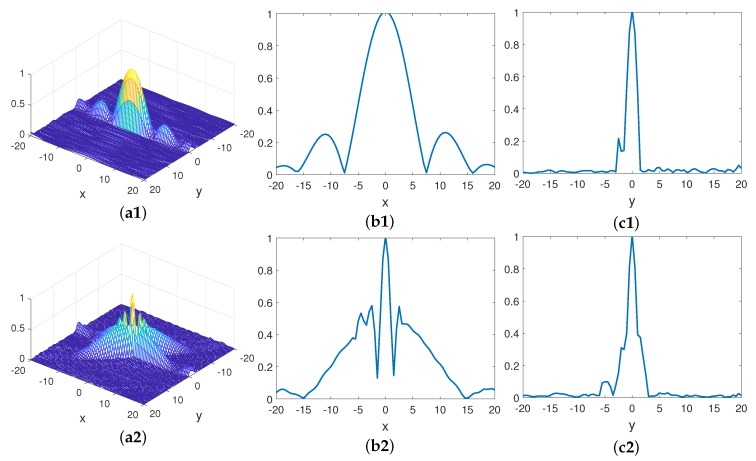
The correlation function of mono-static and bi-static MSCI. (**a**1,**a**2) The space correlation function of mono-static and bi-static MSCI; (**b**1,**b**2) the X-axis profile of the space correlation function of mono-static and bi-static MSCI; (**c**1,**c**2) the Y-axis profile of the space correlation function.

Some parameters are given in Table 4, while the other parameters are the same as those in Table 1. The space correlation functions are computed using (4) and (7); $R({\vec{r}}_{j},\Delta \vec{r})$ of mono-static and bi-static MSCI are illustrated in Figure 9; Figure 9b1,b2,c1,c2 are the *X*-axis and *Y*-axis profiles, respectively.

It can be seen from Figure 9 that the space correlation function of mono-static MSCI is sharp in the *Y*-axis and wide in the *X*-axis and has high side lobe along the *X*-axis, while the space correlation function of bi-static MSCI is sharp in both the *X*-axis and *Y*-axis. However, it has high side lobes along the *X*-axis and the vector (1,−1,0). Additionally, the above results coincide with the analysis in Section 3, that is, the 3 dB beam width of bi-static MSCI is sharper than that of the mono-static MSCI.

### 4.3. The Space Correlation Function in Different Imaging Distances

It can be derived from (18) that the 3 dB beam width of the space correlation function of bi-static MSCI has no relations with the imaging distance, so the following simulation is taken to verify this.

**Figure 10:**
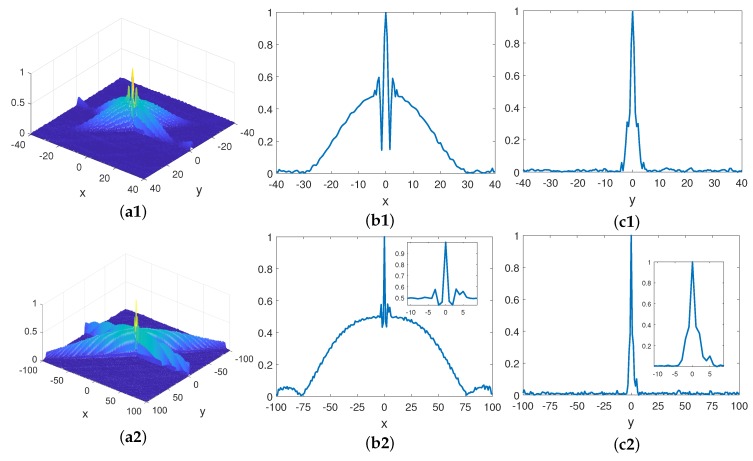
Space correlation function of bi-static MSCI under different imaging distance. (**a**1,**a**2) The space correlation function of bi-static MSCI under different imaging distance 2.03 km and 10.1 km; (**b**1,**b**2) the X-axis profile of the space correlation function of bi-static MSCI under different imaging distance 2.03 km and 10.1 km; (**c**1,**c**2) the Y-axis profile of the space correlation function of bi-static MSCI under different imaging distance 2.03 km and 10.1 km.

The size of imaging area is given in Table 5 and the other parameters are the same as those in Table 1. The heights of the radar are set as 2 km and 10 km. The space correlation function of bi-static MSCI is illustrated in Figure 10. It can be seen from Figure 10b1,c1 and the partial enlarged drawing of Figure 10b2,c2 that the 3 dB beam width of the space correlation function does not change with the imaging distance, while the width of side lobe becomes wider with the increase in the imaging distance, which is in agreement with (18).

### 4.4. The Imaging Capacity with the Number of the Transmitters

The key point of TSSRF is to make the field within the beam independent from each other, thus to acquire super resolution. In practice, the TSSRF is achieved using multi-transmitters transmitting independent radiation field, and the number of transmitters is strongly related with the stochastic characteristic of the radiation field. Reference [13] proved by simulation that the imaging errors decrease with the increase in the number of transmitters when the array aperture and the bandwidth are fixed. However, it can be seen from (18) that the beam width of the space correlation function of the proposed method has a weak relationship with the number of transmitters. Thus, it is necessary to study whether the method can maintain the imaging quality while decreasing the number of transmitters.

Therefore, the below simulation is presented. The NMSE of the imaging results of mono-static and bi-static MSCI is compared by changing the number of transmitters under different imaging distances. The parameters of the imaging area are the same as those in Table 3. The imaging distances vary from 0.5 km to 10 km and 10 individual experiments are taken at each distances.

**Figure 11:**
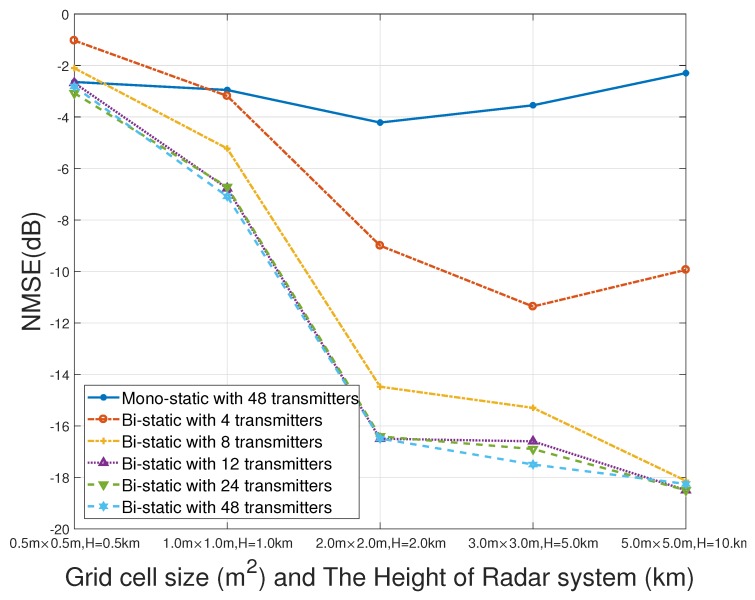
NMSEs of the imaging results by mono-static and bi-static MSCI at different imaging distances and different grid sizes.

The NMSE of different number of transmitters of mono-static and bi-static MSCI is illustrated in Figure 11. It can be seen that the NMSE of bi-static MSCI is much better than mono-static MSCI when there are 48 transmitters. When the number of transmitters decreases while being still greater than 12 (each station has 6 transmitters), the NMSE of bi-static MSCI changes slightly. When the number of transmitters decreases and amount to less than eight, the NMSE of bi-static MSCI decreases greatly while still being better than that of mono-static MSCI.

In brief, decreasing the number of the transmitters will degrade the image quality, which is due to the degeneration of the stochastic characteristic of the radiation field. However, as a result of the superiority of the proposed bi-static imaging geometry, when the number of transmitters decreases, the stochastic characteristic of the radiation field decreases slowly, thus the image quality is maintained. In a practical system, the system complexity needs to be taken into comprehensive consideration. On the premise of ensuring image quality, fewer transmitters can be used to effectively reduce the system complexity.

### 4.5. The Resolution Capability with the Imaging Distance

The 3 dB beam width of the space correlation function of bi-static MSCI does not change with the increase in the imaging distance, while the side lobe becomes wider. Therefore, the resolution capacity of bi-static MSCI cannot be directly concluded. Thus, the following simulations are presented.

In order to measure the resolution capacity, the following definition is given:

**Definition** **1.** *The images are evaluated as accurate recovery under noiseless condition when the NMSE of the imaging results satisfies:*

(21) $$NMSE_{dB}=10\mathrm{lg}\left[{∥\hat{\sigma }-\sigma ∥}_{2}/{∥\sigma ∥}_{2}^{2}\right]<-40dB.$$

**Definition** **2.** *The images are evaluated as basic recovery under noiseless condition when the NMSE of the imaging results satisfies:*

(22) $$NMSE_{dB}=10\mathrm{lg}\left[{∥\hat{\sigma }-\sigma ∥}_{2}/{∥\sigma ∥}_{2}^{2}\right]<-10dB.$$

The radar parameters are given in Table 6 and other parameters are the same as those in Table 1. The algorithm is still the PC-SBL algorithm.

**Figure 12:**
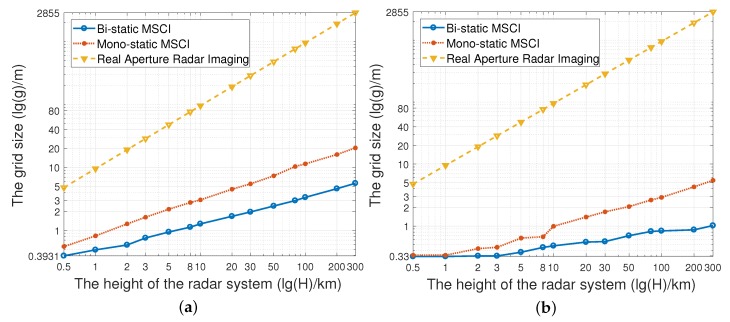
(**a**) The least grid size that satisfies the basic recovery of mono-static and bi-static MSCI under different imaging distances. (**b**) The least grid size that satisfies the accurate recovery of mono-static and bi-static MSCI under different imaging distances.

The least grid size that satisfies the basic recovery and accurate recovery of mono-static and bi-static MSCI under different imaging distances is obtained by increasing the size of the imaging grid step by step in the simulations, the curves are drawn in Figure 12. The curve of the real aperture radar is calculated by $R_{RAR}=\frac{\lambda }{D}L$, where *D* is the array aperture and *L* is the imaging distance and λ is the wavelength of the signals, which is not obtained using simulations.

As the height of the radar platform increases, that is to say, the imaging distance increases, the resolution of the real aperture radar increases linearly, while the least grid size that satisfies the basic recovery and accurate recovery of bi-static MSCI grows much less than the RAR. Under noiseless conditions, the bi-static MSCI can reserve the accurate recovery capacity of 2.9 m and the basic recovery capacity of 1 m while the imaging distance is 50 km. Thus, this proves the effectiveness and superiority of the proposed method.

## 5. Conclusions

In this paper, a novel MSCI method based on a bi-static radar system is proposed, considering both waveform design and bi-static site-deploying. The proposed method adopts two radar stations observing the imaging region from different azimuth angles which differ by 90 degrees. At the same time, the IAIP-FH signals are transmitted to form a latticed radiation field, therefore the stochastic characteristic of the radiation field is partially preserved as the imaging distance increases. The 3 dB beam width of the space correlation function of the radiation field of the bi-static MSCI does not change with the imaging distance. Simulations prove that the proposed method can obtain better imaging results and has a greatly improved resolution capability.

## Figures and Tables

**Table 1:** The simulation parameters of mono-static and bi-static MSCI.

Parameters	Mono-Static MSCI	Bi-Static MSCI
The aperture size	1.6 m × 3.2 m	each array 1.6 m × 1.6 m
The number of receiver	1	1
The carrier frequency	9.2 GHz	9.2 GHz
Bandwidth	500 MHz	500 MHz
The squint angle	$10^{\circ }$	$10^{\circ }$
The pulse period	10 ms	10 ms
The number of pulse	3000	3000

**Table 2:** Parameters of the inner-and-inter pulse frequency hopping (IAIP-FH) signal in this subsection.

Parameters	Mono-Static MSCI	Bi-Static MSCI
Pulse width	400 ns	400 ns
The inner-pulse frequency hopping (FH) interval	10 ns	10 ns
Code number of inner-pulse FH	40	40

**Table 3:** The normalized mean square error (NSME) of the reconstructed images.

Grid Cells Size	The Height of the Radar Platform	Mono-Static MSCI	Bi-Static MSCI
0.5 m × 0.5 m	500 m	−2.87 dB	−2.95 dB
1 m × 1 m	1 km	−3.14 dB	−7.09 dB
2 m × 2 m	2 km	−4.03 dB	−18.03 dB
3 m × 3 m	5 km	−3.39 dB	−15.86 dB
4 m × 4 m	10 km	−2.32 dB	−9.68 dB

**Table 4:** Simulation parameters in this subsection.

Parameters	Mono-Static MSCI	Bi-Static MSCI
Pulse width	200 ns	200 ns
The inner-pulse FH interval	5 ns	5 ns
The height of radar platform	1 km	1 km

**Table 5:** Parameters of imaging distance and imaging region in this subsection.

Parameters	Imaging Distance 1	Imaging Distance 2
Imaging distacne	2.03 km	10.1 km
The size of the imaging region	80 m × 80 m	200 m× 200 m

**Table 6:** Parameters of the transmitted signals in this subsection.

Parameters	Mono-Static MSCI	Bi-Static MSCI
Pulse width	500 ns	500 ns
The inner-pulse FH interval	10 ns	10 ns

## Abbreviations

The following abbreviations are used in this manuscript:

CP	correlation process
cELC	complementary-electric-inductor-capacitor
FH	frequency hopping
IAIP-FH	inner-and-inter pulse frequency hopping
MSCI	microwave staring correlated imaging
RAR	real aperture radar
RD	Range-Doppler
RRS	random radiation source
SAR	synthetic aperture radar
TSSRF	temporal–-spatial stochastic radiation field
TSDE	temporal–-spatial distribution entropy
WGN	white Gaussian noise
